# How to collect non-medical data in a pediatric trial: diaries or interviews

**DOI:** 10.1186/s13063-019-3997-9

**Published:** 2020-01-07

**Authors:** Anaïs Le Jeannic, Hassani Maoulida, Sophie Guilmin-Crépon, Corinne Alberti, Nadia Tubiana-Rufi, Isabelle Durand-Zaleski

**Affiliations:** 1 AP-HP, Groupe hospitalier Hôtel-Dieu, URC Economie de la Santé Ile de France, Paris, France; 2 Inserm, ECEVE UMR-S 1123, Paris, France; 3 URC Eco IdF (Paris health economics and health services research unit) and Inserm, ECEVE UMR-S 1123, Paris, France; 4 AP-HP, Hôpital Universitaire Robert Debré, Unité d’Epidémiologie clinique, Paris, France; 5 Université Paris Diderot, PRES Sorbonne Paris Cité, Paris, France; 6 AP-HP, Hôpital Robert Debré, Service d’Endocrinologie-Diabétologie pédiatrique et Centre de référence des Maladies Endocriniennes Rares de la Croissance, Paris, France; 7 CIC-EC 1426, Paris, France; 8 AP-HP, Groupe hospitalier Albert Chenevier- Henri Mondor, Service de Santé Publique, Créteil, France; 9 Inserm METHODS CRESS UMR 1153, Paris, France

**Keywords:** patient diary, data collection, time costs, investigator-led interview

## Abstract

**Background:**

Non-medical data, such as the amount of time that patients and caregivers spend managing their condition, may be relevant when assessing therapeutic strategies. For chronic pediatric conditions, the time that patients and caregivers spend in seeking and providing care (which are the indirect costs in an economic evaluation) can be significantly different depending on the treatment arm. To explore methods for collecting information on the care burden for caregivers and patients, we investigated whether a patient diary provided additional information compared to retrospective investigator-led interviews and whether a diary that was completed intermittently produced more or less information than a diary completed continually. The main objective of this study was to identify which type of data collection was most effective for measuring the time spent by caregivers and for estimating indirect treatment costs over 9 months.

**Methods:**

Start-In! is a randomized controlled trial comparing the efficacy of three strategies of real-time continuous glucose monitoring for 12 months in children and adolescents with type 1 diabetes. We designed an ancillary study to assess methods of collecting information on the time spent by patients and caregivers in managing their condition (indirect costs). Data were entered retrospectively in case report forms (CRFs) by investigators during quarterly follow-up visits, which were supplemented with diaries completed prospectively by children or caregivers either continuously or intermittently. Data about absences from school and work as well as the time that caregivers spent on diabetes care were collected and the three collection methods were compared.

**Results:**

At the end of the 9-month study, 42% of the study participants failed to return their diary. For the diaries that were received, less than 10% of expected data were collected versus 82% during investigators'interviews. Based on all the information collected, we calculated that over 9 months, caregivers lost on average 3.9 days of working time (€786) and 4 days of personal time, i.e. the equivalent of €526, and spent around 15 min of time on care per day, i.e. the equivalent of €1700.

**Conclusions:**

The CRFs completed by investigators during quarterly visits cannot be replaced by a diary. Completing the diaries appeared to represent an important additional burden to children and their caregivers, and the diaries provided little additional information compared to investigators’ entries in the CRF.

**Trial registration:**

ClinicalTrials.gov, NCT00949221. Registered on 30 July 2009. Registry name: Study of Insulin Therapy Augmented by Real Time Sensor in Type 1 Children and Adolescents (START-IN!).

## Background

Non-medical data, such as the time spent by patients and caregivers in managing their condition, may be relevant in assessing the full burden and cost of a therapeutic strategy. The pertinence of including time costs for caregivers and patients as indirect costs in economic evaluations of therapeutic strategies may be considered when writing the protocol and mainly depends upon the perspective of the evaluation and the type of illness being considered [1]. Productivity losses can represent a significant amount of resources, especially for chronic pediatric conditions because caregivers may need to seek and also provide care for children. Information on absences from work or school is not recorded in health insurance databases, so must be estimated, either through retrospective interviews with an investigator [2] or from diaries completed by the parents or patients [3, 4]. Investigator-led interviews require additional work from the physicians, which could be reduced if the children or their caregivers completed diaries prospectively [5]. However, the use of paper or electronic diaries in clinical trials requires logistical management and fastidiousness from participants, especially for studies with long follow-up periods [6]. This may be more problematic for pediatric trials, since parental constraints may influence the acceptance of additional methods of data collection [7].

To determine the best way to collect information on care burden and time costs, we investigated whether or not a patient-completed diary would provide additional information compared to retrospective investigator-led interviews and whether a diary completed intermittently would produce more or less information than a continuous diary. We hypothesized first that a diary would enable investigators to shorten their interviews, which would be helped by or even replace the diaries. We assumed that patients would tend to underreport their time costs. Our second hypothesis was that keeping a continuous diary would be an onerous task and could lead to fatigue and poor data collection over time. However, the intermittent diary, while reducing the data entry burden for patients [8], has the risk that patients may fail to complete it if it was not a regular task.

The main objective of this study was to identify which type of data collection method was most effective for obtaining information on care burden and time costs over the 9 months of a pediatric trial. The secondary objectives were to determine the patient acceptance of an intermittent versus a continuous diary and to check whether the amount of data collected decreased over time.

## Methods

### Study design

Start-In! was a French multi-center trial with a randomized controlled prospective open parallel-group design, comparing three therapeutic modalities in pediatric management [9]. We designed an ancillary study to the Start-In! trial (NCT00949221) that compared the efficacy and cost-effectiveness of three strategies of real-time continuous glucose monitoring (RT-CGM) for children with type 1 diabetes [9]. This trial was well adapted to our study objective since insulin-dependent diabetes in children is a chronic condition with major time costs to children and their parents.

**Fig. 1:**
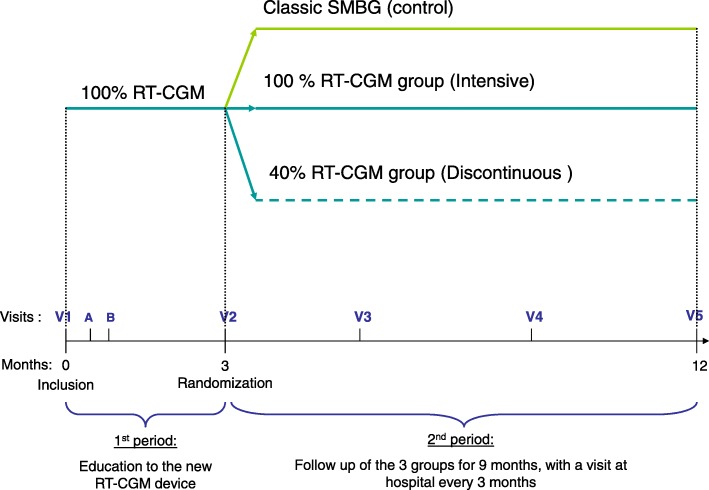
Design of the Start-In! trial. The discontinuous group used intermittent RT-CGM alternating with SMBG. RT-CGM real-time continuous glucose monitoring, SMBG self-monitoring blood glucose

The patients in Start-In! were aged from 2 to 17 years. They had been diagnosed with type 1 diabetes for more than 1 year and were being treated by intensive insulin therapy, achieving inadequate metabolic control. The study was conducted in 11 pediatric diabetology units with expertise in pump therapy and CGM. The primary endpoint was long-term glycemic control at 12 months. The clinical study had two phases: all subjects wore glucose monitors during the first 3 months, and thereafter were randomized into three groups with different strategies for glucose monitoring during the next 9 months. The ancillary study reported here concerns the 9-month follow-up period of the patients (Fig. 1).

The Start-In! economic evaluation required information on consultations, absences from work or school due to diabetes, and the time spent by the caregiver on average each day on providing diabetes care. This information was entered for all patients by the investigators in paper case report forms (CRF) at the end of each trimester and represented the reference strategy.

**Fig. 2:**
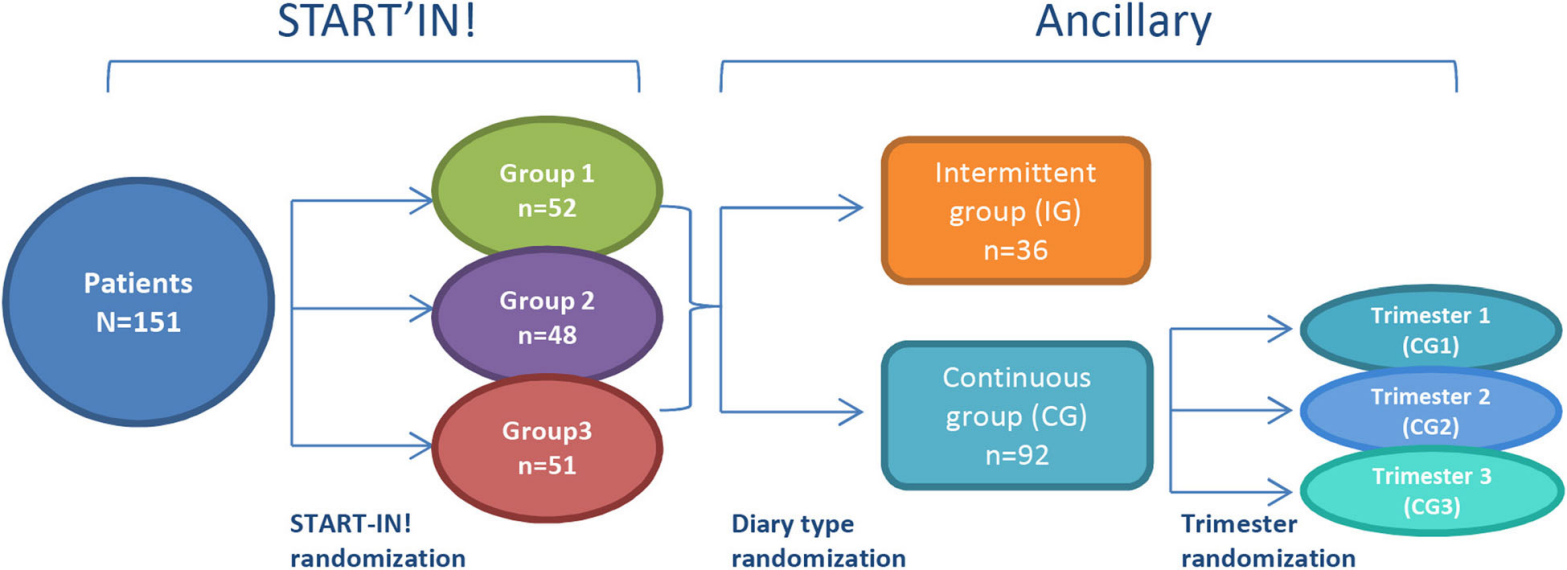
Groups for the Start-In! trial and the present study. Groups 1, 2, and 3 in the Start-In! trial used classic SMBG, 100% RT-CGM, and 40% RT-CGM, respectively. CG continuous group, IG intermittent group, RT-CGM real-time continuous glucose monitoring, SMBG self-monitoring blood glucose

**Fig. 3:**
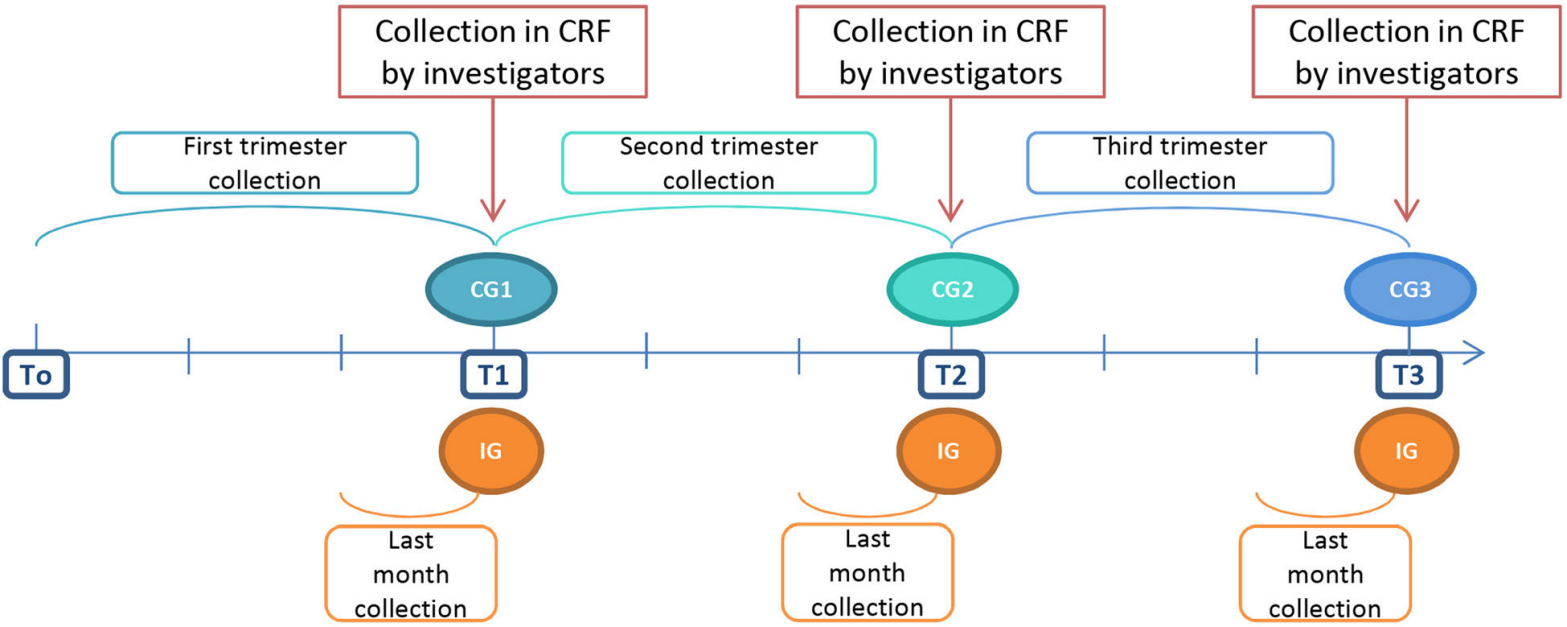
Timeline of data collection for each randomization group. T0 marks the end of the third month of Start-In! Blue ovals represent the continuous groups (CG1, CG2, and CG3) and orange circles represent the intermittent group (IG). CG, continuous group, CRF case report form, IG intermittent group

To test whether diaries would provide additional information, patients were randomized into two groups. In the continuous group, children and caregivers were asked to collect data in a diary without interruption during one of the three trimesters of the study (3 months continuously). In the intermittent group, data were collected only during the last month of each of the three trimesters. Randomization was in a 3:1: ratio. In the continuous group, the diary was completed daily for one of the three trimesters of the study follow-up period, allocated randomly in a 1:1:1 ratio. To simplify the work for the investigators, the randomization for the ancillary study was by center and not by patient (Figs. 2 and 3). Data from the patient interviews and diaries were entered into the CRFs by investigators following the same schedule: one trimester for the continuous group and the last month of each trimester for the intermittent group.

### Endpoint

The primary endpoint was the amount of information recorded in the diaries, compared to the amount of information given to the investigator during the quarterly follow-up visit. The Start-In! participants (patients and caregivers) were asked to record in their diary: (1) for the child, the dates of each period of absence from school or work, (2) for an adult caregiver, the dates of each period of absence from work, (3) the date of each consultation, (4) the number of caregivers present at each consultation, and (5) an estimate of the time spent on average each day by the caregivers to provide diabetes care such, as blood glucose monitoring and insulin administration (Additional file 1). There was no minimum or maximum for the number of absences or visits that could be recorded. The same information was also collected quarterly by the investigators and entered in the CRF with the help of the diary, which was collected at the same time.

### Analysis

The number of absences from work or school and the number of consultations were calculated from the data entered in the diaries for each patient. If the amount of time spent by the caregiver on average each day was not completed, it was considered to be missing data rather than zero. If the other data items were not completed at all (dates of absences from school or work, and dates of consultations), we considered that the data were missing if the average time spent by the caregiver was also missing. The overall amount of information recorded was compared between the intermittent and continuous groups, and with the amount of information entered by the investigators into the CRFs.

#### Care burden and indirect costs

For the indirect costs in the economic evaluation, the opportunity cost was based on the number of absences from work or school for the patients and their families. For those who were in employment, it was based on the average salary in France (€34 per hour) and for those who were unemployed, on the average salary of a house cleaner (€18.7 per hour). The average daily time spent by a caregiver administering diabetes care to a patient was valued based on the average salary of a home nurse (€28.8 per hour). All costs are in euros (€) for 2017 (1€ = 1.2US$) and were not discounted due to the short time horizon.

## Results

A total of 151 patients were randomized in the Start-In! trial, 52 into Group 1, 48 into Group 2, and 51 into Group 3. Their characteristics were similar at inclusion [9]. They had an average age of 12 years and there was nearly a 50:50 ratio of boys to girls, although there was large differences in age and sex between the centers, as seen in Table 1. A total of 23 patients who left the study were excluded from our ancillary analysis of the diary data, as their diaries would, obviously, be empty. Thus, diaries were expected from 128 patients for analysis, 92 in the continuous group and 36 in the intermittent group. Information on care burden was additionally collected on all 151 patients by the investigators during the follow-up visits. However, almost half of the 128 patients in this analysis did not return a single diary, so the information they reported to the investigators and entered in the CRF was based solely on their memory.

**Table 1:** Frequency, mean age, and ratio of boys to girls at inclusion

Centre	Frequency	Mean age (years)	Sex ratio (boys/girls)
1	30	11.1	1.14
2	31	11.2	2.88
3	17	11.8	1.43
4	9	12.3	0.80
5	19	13.9	0.36
6	13	10.9	0.44
7	9	14.2	0.50
8	8	11.7	1.67
9	6	9.8	0.50
10	6	11.5	2.00
11	3	12.5	0.50
Overall	151	11.8	1.04

### Continuous group

Of the 92 patients, only 53.3% (*n* = 49) returned their diary. Of the 49 diaries returned, 44 were empty so data were considered to be missing. Four diaries reported that no absences had occurred and only one diary gave the date of an absence from work or school. That is, 89.8% of the diaries received had missing data. Overall, of the 92 diaries expected, 99.5% were either not received or had missing data.

### Intermittent group

Of the 36 patients, just under half (*n* = 16) returned at least one diary. Each patient should have submitted three diaries, one for each trimester, but only 41 of the 108 diaries expected were received. Of those 41 diaries, 97.6% had missing data, as only one diary was complete. Overall, 99.5% of diaries from patients were either not received or had missing data.

### Investigator-completed CRF

The same information was collected retrospectively from the patients and caregivers by investigators, who entered it into the CRFs at the trimestral follow-up visits. The information was, potentially, supplemented by the data entered prospectively in the diaries.

Table 2 shows the numbers of CRFs completed for each data item by the investigators for patients whose diaries were missing or empty, by randomization group (continuous or intermittent). For example, for the continuous group, regarding *absences from work*, among the 87 patients who either returned an empty diary or no diary, this information was recorded for 73 in their CRF. For the *consultations* section, among the 35 patients from the intermittent group with empty or no diaries, this information was recorded for 34 in their CRF. We calculated the percentage of data missing from the CRFs in the same way we did for diaries. The continuous group had more data missing in their CRFs than the intermittent group (14% vs. 5%), which is far less than the around 99% of data missing from the diaries. In conclusion, it appeared that for the diaries that were received, less than 10% of expected data were collected versus 82% during investigators'interviews.

**Table 2:** Frequency of CRFs completed for each data item for patients not returning diaries or returning empty diaries

Data item	Continuous group, *n* (%) *N* = 87	Intermittent group, *n* (%) *N* = 35
Absences from school	78 (90)	34 (97)
Absences from work	73 (84)	32 (91)
Consultations	81 (93)	34 (97)
Time spent on diabetes care	68 (78)	33 (94)

### Care burden and indirect costs

Since the CRFs, which were based upon interviews and diaries, held about 80% of the indirect cost data, we calculated productivity losses and indirect costs. Over 9 months, parents lost on average 3.9 days of working time, i.e. the equivalent of €786, and 4 days of unemployed time, i.e. the equivalent of €526, and spent around 15 min of time on care per day, i.e. the equivalent of €1700. Children were absent from school for an average of 3 days over the 9 months.

## Discussion

Some trials require non-medical data that are not readily available from patients’ charts or an insurance database. Investigators must either rely on patients’ recollection at the time of a follow-up visit or provide diaries. Our objective was to estimate the indirect medical costs in each treatment arm and identify possible differences that cannot be captured by calculating direct medical costs based upon information systematically retrieved from hospital insurance claims data. All investigators were asked to record in the CRFs data on caregiver and patient burden (time), and they relied on information obtained from both interviews and diaries. Our study compared two prospective methods of documenting the treatment burden for caregivers and children with type 1 diabetes during a clinical trial, which included an economic evaluation. Our major finding was the magnitude of the data missing in the diaries provided to caregivers (around 99%). While the average cost of caregivers’ time could be estimated from investigator-led interviews to be around €3000 per patient over 9 months, the information from diaries alone did not allow a reliable estimate to be calculated. It would appear that asking families to record non-medical information in a diary during a pediatric trial of a severe chronic condition is infeasible, and we do not recommend their use.

The amount of data entered by investigators for patients in the intermittent group was slightly higher than for the continuous group (95% vs. 86%). This may be because the participants were asked to remember information for only one month at the time of an interview instead of for three [10]. In this trial, the CRFs always contained more data than the diaries since the CRFs were based upon interviews supplemented by the diaries (if the diaries had data). In fact, diaries can be a reliable source of data to complement usual reporting methods, as shown by Dunn et al. [5], but we were not able to confirm this conclusion because of the amount of missing diary data.

Many studies from different countries show a high concordance between self-reported and medical records of healthcare resource utilization in the general patient population [11, 12]. We were interested in pediatric trials, in which the diaries would be completed by caregivers, who may be tired from caring for a chronically ill child in addition to their jobs and may have other children to take care of. In fact, family members of someone with diabetes seems to be at higher risk of depression [13] and parents of a child with type 1 diabetes not only struggle with depression [14, 15] but also worry about hypoglycemia [16], which can lead to parental emotional distress [17]. Difficulties are encountered with adolescent patients, who are becoming more independent but have troubled relationships with their parents [18] and may have transition issues, like treatment adherence [19, 20]. In those circumstances, reporting non-medical data, such as school absences, may not be a priority for the family.

Unfortunately, the patients and their caregivers were not asked to give their opinions about the different methods of data collection, so we can only speculate about why so much data were missing. This study has other limitations. The amount of missing data prevented us from performing statistical tests or evaluating our secondary objectives of patient acceptance and fatigue. Moreover, with such little data, the quality of the information provided in the diaries could not be assessed and thus, only the quantity was explored in our trial.

## Conclusion

Trials that require non-medical data may need to rely on patients and families for information. In our study of a pediatric chronic condition, investigator-led interviews provided much more information than patient diaries. The completion of diaries appears to represent an important additional burden to children and their families, who are already struggling with many competing issues. Notwithstanding, the large indirect costs associated with the loss of parental productivity underlines the importance of collecting these data, in one way or another.

## Supplementary information

**Additional file 1.** Data collected by patients in the diaries and also in the CRFs.

## Data Availability

The datasets analyzed during the current study are available from the corresponding author on reasonable request.
