# Supplementary material for: How to collect non-medical data in a pediatric trial: diaries or interviews
Source: Trials. 2020 Jan 7;21:36. doi: 10.1186/s13063-019-3997-9 (PMC6947947; doi:10.1186/s13063-019-3997-9)
Supplement: Supplementary file 1 — Additional file 1. Data collected by patients in the diaries and also in the CRFs. [file 13063_2019_3997_MOESM1_ESM.pdf]

Additional file 1: Data collected by patients in the diary.ies and CRF.

Economic evaluation:

Note here any diabetes-related absences during the ....° month of this trimester:

- Patient's days of absence from school (or work):

from....../....../...... to ....../....../...... included
from....../....../...... to ....../....../...... included
from....../....../...... to ....../....../...... included
from....../....../...... to ....../....../...... included

- Days of absence from work of an adult (father, mother...):

from....../....../...... to ....../....../...... included
from....../....../...... to ....../....../...... included
from....../....../...... to ....../....../...... included
from....../....../...... to ....../....../...... included

Note here the medical consultations (for diabetes or not) carried out during the....° month of this quarter:

Consult n°1 : the .../........./...........
........attending adults

Consult n°2: the...../........./...........

........attending adults

Consult n°3 : the..../........./...........
........attending adults

Consult n°4 : the..../........./...........
........attending adults

Consult n°5 : the..../........./...........
........attending adults

Note here your estimate of the time spent during 24 hours ("typical" day) at home, by the father, mother and/or other adult, for diabetes treatment and care:

NB: - treatment care, continuous glucose measurement, on the internet or by phone for analysis and advice.

- do not count the time spent alone by the patient.

Date: ..../........./.........

Morning: ....h.........
Noon: ....h.........
Afternoon: ....h.........
Evening: ....h.........
Night: ....h.........
